# Radiotherapy for HPV-related cancers: prediction of therapeutic effects based on the mechanism of tumor immunity and the application of immunoradiotherapy

**DOI:** 10.1007/s11604-021-01231-4

**Published:** 2022-01-01

**Authors:** Masanori Someya, Yuki Fukushima, Tomokazu Hasegawa, Takaaki Tsuchiya, Mio Kitagawa, Toshio Gocho, Shoh Mafune, Yutaro Ikeuchi, Yoh Kozuka, Yoshihiko Hirohashi, Toshihiko Torigoe, Masahiro Iwasaki, Motoki Matsuura, Tsuyoshi Saito, Koh-ichi Sakata

**Affiliations:** 1 Department of Radiology, Sapporo Medical University School of Medicine, Chuo-ku, Sapporo, Hokkaido 060-8543 Japan; 2 Department of Pathology, Sapporo Medical University School of Medicine, Sapporo, Japan; 3 Department of Obstetrics and Gynecology, Sapporo Medical University School of Medicine, Sapporo, Japan

**Keywords:** Cervical cancer, Radiotherapy, Tumor immunity, CD8, Forkhead box P3, Human leukocyte antigen class I, Programmed death ligand 1, CD8 based subtyping

## Abstract

Human papillomavirus (HPV)-related cancer is one of the diseases entities for which the applications of radiotherapy have been increasing. Recently, the process of carcinogenesis from HPV infection and the mechanism of tumor immunity that develops during disease progression have been elucidated. In this review, we will describe the mechanism of tumor immunity and how chemoradiotherapy may overcome and improve the efficacy of tumor immunity. We will also discuss the usefulness of proteins involved with tumor immunity as a predictive marker of radiotherapy response, and present an overview of ongoing clinical trials of combinations of immune checkpoint inhibitors and radiotherapy to demonstrate the promising combination therapy that has been currently emerging.

## Introduction

Human papillomavirus (HPV)-related cancer is one of the diseases entities for which the applications of radiotherapy have been increasing. In this review, we will describe the mechanism of tumor immunity and how chemoradiotherapy may overcome and improve the efficacy of tumor immunity, and present an overview of ongoing clinical trials of combinations of immune checkpoint inhibitors and radiotherapy.

## Human papillomavirus (HPV) infection and the development of HPV-related cancer

HPV is a double-stranded cyclic DNA virus belonging to the human papillomavirus family that targets and infects epithelial cells of the skin, oral cavity, cervix, and anus. [1]. The viral genome consists of three regions: the early E regions (E1, E2, E4, E5, E6, and E7), the late L regions (L1 and L2), and the upstream regulatory region (URR). [1, 2]

Among HPV-related cancers, a lot of data has been reported on cervical cancer. According to the International Agency for Research on Cancer, 12 subgroups of HPV (16, 18, 31, 33, 35, 39, 45, 51, 52, 56, 58, and 59) are considered high risk. High-risk HPV types are responsible for cervical, vulvar, penile, anal, and oropharyngeal cancers [3], especially about half of the oropharyngeal cancers and about 95% of cervical cancers are caused by the high-risk HPV infection [4]. The majority of HPV infections are confined to the epithelial cells, and approximately 90% of infections result in the elimination of the virus mediated by humoral immunity within a few months [5, 6]. Persistent infection occurs in the remaining 10% of patients and increases the risk of cancer development in about 1% of patients [6].

After the infection, the early HPV genes (E1-7) are first expressed. In the upper layers of epithelial cells, the viral genome is replicated and the late genes (L1 and L2) and E4 are expressed to assemble the viral particles. Normally, epithelial cells lose their nucleus and the ability to proliferate when they differentiate into keratinocytes, however, HPV uses the host's DNA replication system for self-renewal, and the E6 and E7 proteins interact in a distinctive manner to keep keratinocytes in a proliferative state. In particular, E7 inactivates the retinoblastoma protein (pRB) to maintain the proliferative state, while E6 degrades p53 to prevent apoptosis [7, 8]. This negative regulation of the cell cycle and the accumulation of the genetic damages caused by the virus-derived oncoproteins promote oncogenesis, which progresses to high-grade squamous epithelial lesions and invasive carcinoma over years after persistent infection.

## Mechanisms of cancer cell escape from tumor immunity

In the process of development of clinically advanced cancer, the immune system suppresses cancer progression by recognizing the tumor as non-self (foreign) and attacking the tumor [9, 10]. In contrast, the tumor microenvironment is composed of a variety of cells that allow cancer cells to escape anti-tumor immunity [11, 12]. As the cancer cells grow, they affect the activation of immune cells in the tumor microenvironment and its components such as the stroma [13]. Although cancer cells are originally derived from normal cells, HPV-associated cancers are more likely to be recognized as nonself due to the high expression of tumor-specific viral neoantigen, and cytotoxic T cells (CTLs) are thought to be more easily activated against cancer cells [9, 14, 15].

When cancer cells are exposed to the innate immune system, such as Natural Killer cells, signaling molecules are released, resulting in an acquired immune response by antigen-presenting cells (APCs) [16, 17]. The acquired immune response consists of the steps of antigen presentation, infiltration, and elimination, and the production of proinflammatory cytokines upon stimulation of APCs activates T cells and promotes tumor inflammation. Acquired immunity is antigen-dependent and antigen-specific, and can lead to a continuous immune response. CTLs, the major effector cells, are activated by non-self, such as cancer antigens [9, 18]. Once activated, CTLs proliferate, migrate to the site of the antigen, infiltrate, and begin to directly attack the cancer cells [19].

Tumors are classified as cold or inflamed types according to the degree of infiltration of immune cells; cold types have few immune cells and are thought to have an impaired ability to present cancer antigens to T cells or to secrete chemokines [12, 20]. The induction of tumor-specific T cells and their infiltration are suppressed, resulting in a failure to eliminate cancer cells [21]. In contrast, the inflamed type is characterized by the presence of immune cells [20–22], more active antigen presentation, and infiltration of CTLs activated by chemokine expression into the tumor microenvironment [21, 23]. However, cancer cells may upregulate the expression of various inhibitory proteins and escape elimination by CTLs [24].

## Immune escape of HPV-related cancer may overcome by radiotherapy

The immune response to radiotherapy is thought to be the result of increased antigen presentation and recognition by the host immune system as a result of tumor cell death [25]. A mechanism underlying the favorable response of HPV-related cancers to chemoradiotherapy is reported that chemoradiotherapy causes cell damage and inflammation, leading to the release of inflammatory cytokines such as IL-6, IL-8, and tumor necrosis factor (TNF)-α, as well as Damage-associated molecular patterns (DAMPs) such as High-mobility group-box 1 (HMGB1), and upregulation of signals that promote phagocytosis by dendritic cells such as calreticulin [26].

HMGB1 recruits dendritic cells and macrophages to promote inflammation and tissue repair. HMGB1 also interacts with dendritic cells via Toll-like receptor 4, promoting dendritic cell maturation and the induction of helper T cell responses [27]. Dying tumor cells also release viral neoantigen, which is captured by dendritic cells. The combination of DAMPs and viral neoantigen generates an immune response strong enough to overcome the immune escape of the tumor [26].

## Prediction of radiotherapy effect in HPV-related cancer based on tumor immunity

Among head and neck cancers, especially in oropharyngeal cancers, it has been known that HPV-related, p16-positive cancer patient have significantly better treatment outcomes than p16-negative patient [28]. Favorable radiosensitivity is also observed in cervical and anal cancers, where the majority of cases are considered to be HPV-related.

The high immunogenicity of HPV-related cancers is a suggested mechanism to explain the radiosensitivity. Radiotherapy is considered to induce immunogenic cell death, resulting in increased antigen presentation, inflammation, and induced dendritic cells, which in turns activates CTLs [29–31].

## Prediction of treatment response by CD8-positive T cell infiltration

CTLs differentiate from T cells that express CD8 molecules on their surface. In naive CD8-positive T cells, which do not possess cytotoxic activity, their T cell receptors (TCRs) recognize cancer antigen peptides presented as class I major histocompatibility antigens (MHC class I) on antigen-presenting cells and simultaneously receive signals from co-stimulatory molecules. At the same time, the cells become CTLs with cell-specific cytotoxic activity, releasing Perforin, Granzyme, and TNF to attack the cancer cells. Our previous studies have shown that cervical and oropharyngeal cancers with an abundant invasion of CD8 + T cells in the tumor in biopsy specimens have better treatment outcomes with chemoradiotherapy, and there have been many other similar reports [32–35]. HPV-derived cancer cell antigens may promote T cell infiltration, induce immune responses, and create an inflammatory microenvironment. [26].

## Prediction of treatment response by FoxP3-positive T cell infiltration

Regulatory T cells (Tregs) are responsible for suppressing immune responses to self (immune tolerance) to prevent autoimmune diseases, and account for about 5% of CD4 positive T cells in healthy individuals. Cancer cells are thought to use these Tregs to escape from attacking by the immune system through reducing the effector T cells. FoxP3 (Forkhead box P3) is well known as a marker of Tregs and has been intensively investigated. It has been reported that in many solid cancers, more invasion of FoxP3 + Tregs is associated with worse prognosis [36], but in HPV-related cancers, several conflicting results have been reported, and the results have not been consistent [37].

Our previous reports have shown that chemoradiotherapy in cervical cancer with high infiltration of FoxP3 + T cells in the stroma around tumor in biopsy specimens showed better outcomes [32], and a similar result has been shown in chemoradiotherapy for anal cancer [38]. In this paper, the authors also reported that a higher degree of infiltration of FoxP3 + Tregs in the tumor correlated with a higher rate of HPV16 positivity and better local control. This suggests that the co-infiltration of CD8 + T cells and FoxP3 + T cells may explain the unexpected positive role of Tregs [38]. Consistent with these findings, we have also shown that in cervical cancer, the number of CD8 + T cells infiltrating the tumor correlates with the number of FoxP3 + T cells [32]. Therefore, at least in HPV-related cancers, FoxP3 + T cells may be a good predictive marker in response to chemoradiation.

## Prediction of treatment response by PD-L1 expression

The interaction between PD-L1 on the surface of tumor cells and its receptor PD-1 expressed by T cells results in suppression of T cell activation and induction of T cell apoptosis [39, 40]. Therefore, PD-L1 expression is considered to be a state reflecting active anti-tumor immunity. In our previous reports, high PD-L1 expression has been found to be a predictive marker for better treatment outcomes in preoperative radiotherapy of cervical cancer and chemoradiotherapy of oropharyngeal cancer. The upregulation of PD-L1 by radiation therapy has been demonstrated in several preclinical models [41, 42]. Recently, it has been reported that induction of double-stranded DNA breaks by radiation upregulates PD-L1 expression via the ATM/ATR/Chk1 pathway [41]. These results suggest that immunotherapy with immune checkpoint inhibitors (ICIs) such as anti PD-1 and anti PD-L1 antibodies combined with radiotherapy will be promising.

## Prediction of treatment response by CD8 + T cell-based subtyping

The concept of T-cell-based tumor classification originated from the observation that the type, density, and location of immune cells within a tumor site can predict colorectal cancer (CRC) survival more accurately than the classical TNM system [43]. Based on these factors, prognosis is estimated by a method called Immunoscore, which proved to be a more useful prognostic tool for patients with CRC [44]. In this classification, cancer patients with more T-cell invasion, described as ‘hot tumor’, are known to have a better prognosis than those with less T-cell invasion, described as ‘cold tumor’ [45, 46]. Since radiotherapy leads to immunogenic cell death, the release of neoantigens, and consequently activation of T cell-mediated immunity [47], this concept has been applied to predict the efficacy of radiotherapy.

**Fig. 1:**
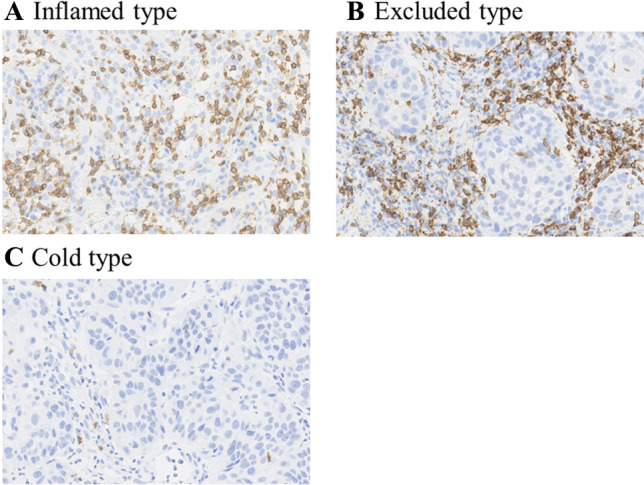
CD8-positive T cell-based classification of the cancer microenvironment. (**A**) Inflamed type: defects in tumor recognition by T cells, (**B**) Excluded type: failure of T cell infiltration into the tumor, (**C**) Cold type: lack of antigen presentation of tumor cells

In our previous results, we divided tumors into inflamed, excluded, and cold types based on the type of CD8 + T-cell infiltration in biopsy specimens prior to chemoradiation of cervical cancer. We found that the treatment results of Inflamed and Excluded types were almost the same, whereas Cold type had a worse prognosis and the tumor volume was predominantly larger in Cold type (Fig. 1 and Table 1), [48]. It is suggested that radiotherapy can overcome the factors that inhibit CD8 + T cells from infiltrating into the tumor [26], and this may be one of the reasons why radiotherapy can enhance the therapeutic effect of subsequent immunotherapy, especially in excluded type tumors.

**Table 1:** Relationship between CD8-based subtyping and tumor immunity

	Cold type	Excluded type	Inflamed type
Tumor volume	+	±	–
CD8	–	+	+ +
FoxP3	–	+	+
HLA-1	–	±	+
PD-L1	–	–	+
Response to radiotherapy	Poor	Better	Better

Cold-type tumors may escape the tumor immune surveillance system, such as tumor antigen presentation and tumor recognition by immune cells, and the formation of hypoxia and abnormal tumor vasculature during tumor growth may make it difficult for immune cells to infiltrate [49, 50]. Therefore, to overcome this type of tumor, the development of therapies such as anti-VEGF antibody therapy to normalize abnormal blood vessels and vaccine therapy to create in situ vaccination in the tumor will be necessary [51].

## The possibility of radio-immunotherapy for HPV-related cancer

The strongest clinical evidence for the potential of combined radiotherapy and ICIs is the recently announced PACIFIC trial [52]. Patients with locally advanced non-small-cell lung cancer who had received standard chemoradiation therapy were randomized to receive the PD-L1 inhibitor durvalumab postoperatively or placebo and a significant prolongation of overall survival was observed in the durvalumab group.

Radiation can change the microenvironment of tumors and turn immunosuppressive tumors into inflamed tumors, which may enhance the therapeutic effect [53]. The development of protocols and further mechanistic investigations in clinical trials, as will be described later, are expected to clarify the combined role of radiotherapy and ICI in HPV-related cancers.

## Clinical trials of CRT and ICI combination for HPV-related locally advanced cancer

Main ongoing clinical trials of CRT and ICI combination for HPV-related locally advanced cancer as of October 2021 are summarized in Table 2 and the main features are described below.

**Table 2:** Clinical trials in HPV-related locally advanced cancer combined with ICI and radiotherapy/chemoradiotherapy

Trial identifier	Title/description	Phase	Cancer site	Estimated enrolled patients	Drug	Primary endopoint	Status	Estimated study completion
NCT03612791	Trial Assessing the Inhibitor of Programmed Cell Death Ligand 1 (PD-L1) Immune Checkpoint Atezolizumab (ATEZOLACC)	2	Cervix	189	Atezolizumab	PFS	Recruiting	2022-07-01
NCT02635360	Pembrolizumab and Chemoradiation Treatment for Advanced Cervical Cancer	R2	Cervix	88	Pembrolizumab	Immunologic markers, DLT	Active, not recruiting	2021-12-01
NCT03527264	Nivolumab to Tailored Radiation Therapy With Concomitant Cisplatin in the Treatment of Patients With Cervical Cancer (BrUOG 355)	2	Cervix	Actual enroll 4	Nivolumab	PFS, AE	Active, not recruiting	2023-12-01
NCT03833479	TSR-042 as Maintenance Therapy for Patients With High-risk Locally Advanced Cervical Cancer After Chemo-radiation (ATOMICC)	2	Cervix	132	TSR-042 (Dorstarlimab)	PFS	Recruiting	2023-07-01
NCT03830866	Study of Durvalumab With Chemoradiotherapy for Women With Locally Advanced Cervical Cancer (CALLA)	3	Cervix	714	Durvalmab	PFS	Active, not recruiting	2024-06-30
NCT04221945	Study of Chemoradiotherapy With or Without Pembrolizumab For The Treatment of Locally Advanced Cervical Cancer (KEYNOTE-A18/ENGOT-cx11/GOG-3047)	3	Cervix	980	Pembrolizumab	PFS, OS	Recruiting	2024-12-07
NCT03799445	Ipilimumab, Nivolumab, and Radiation Therapy in Treating Patients With HPV Positive Advanced Oropharyngeal Squamous Cell Carcinoma	2	OPSCC	180	Ipilibumab+Nivolumab	DLT, CR rate, PFS	Recruiting	2022-08-01
NCT03715946	Adjuvant De-Escalated Radiation + Adjuvant Nivolumab for Intermediate-High Risk P16+ Oropharynx Cancer	2	OPSCC	actual enroll 42	Nivolumab	PFS, PEG dependance	Active, not recruiting	2023-12-30
NCT04230759	Radiochemotherapy +/− Durvalumab for Locally-advanced Anal Carcinoma. A Multicenter, Randomized, Phase II Trial of the German Anal Cancer Study Group (RADIANCE)	R2	Anal	178	Durvalumab	DFS	Recruiting	2026-06-30

*HPV* Human papillomavirus, *ICI* Immune checkpoint inhibitor, *PFS* Progression-free survival, *DLT* dose-limiting toxicities, *AE* Adverse events, *OS* Overall survival, *PEG* percutaneous endoscopic gastrostomy, *DFS* Disease-free survival, *OPSCC* Oropharyngeal squamous cell carcinoma

ATEZOLACC (NCT03612791) is a randomized phase II trial assessing the benefit of atezolizumab combined with standard CRT for cervical cancer. Atezolizumab is administered every 3 weeks for a maximum of 20 cycles.

Pembrolizumab and Chemoradiation Treatment for Advanced Cervical Cancer (NCT02635360) is a randomized phase II trial combined with CRT and Pembrolizumab for the treatment of cervical cancer.

BrUOG 355 (NCT03527264) Nivolumab to Tailored Radiation Therapy With Concomitant Cisplatin in the Treatment of Patients With Cervical Cancer. This phase II clinical trials has 3 different arms to test the safety and effectiveness of combination with CRT and Nivolumab at 3 different timing, during CRT, maintenance after CRT, and both during and maintenance after CRT.

ATOMICC (NCT03833479) TSR-042 as Maintenance Therapy for Patients With High-risk Locally Advanced Cervical Cancer After Chemo-radiation. A randomized phase II trials designed to use of 24 months of Dostarlimab (TSR-042), checkpoint inhibitor, as consolidation therapy following concurrent CRT for cervical cancer.

CALLA (NCT03830866)Study of Durvalumab With Chemoradiotherapy for Women With Locally Advanced Cervical Cancer. This is a randomized, multi-center, double-blind, placebo-controlled, global, phase III study to determine the efficacy and safety of durvalumab plus CRT versus CRT alone as treatment in locally advanced cervical cancer.

KEYNOTE-A18/ENGOT-cx11/GOG-3047 (NCT04221945) Study of Chemo-radiotherapy With or Without Pembrolizumab For The Treatment of Locally Advanced Cervical Cancer. The purpose of this randomized phase II study is to evaluate the efficacy and safety of 15 cycles of pembrolizumab plus concurrent CRT compared to placebo plus concurrent CRT in patients with locally advanced cervical cancer.

Ipilimumab, Nivolumab, and Radiation Therapy in Treating Patients With HPV Positive Advanced Oropharyngeal Squamous Cell Carcinoma (NCT03799445). This phase II trial studies the side effects and best dose of ipilimumab, nivolumab, and RT for patients with HPV positive oropharyngeal squamous cell carcinoma.

Adjuvant De-Escalated Radiation + Adjuvant Nivolumab for Intermediate-High Risk P16 + Oropharynx Cancer (NCT03715946). This clinical trial will evaluate a combination of standard CRT and nivolumab for p16 positive oropharyngeal squamous cell cancers.

RADIANCE (NCT04230759) Radiochemotherapy ± Durvalumab for Locally advanced Anal Carcinoma. A Multicenter, Randomized, Phase II Trial of the German Anal Cancer Study Group. This multicenter, randomized phase II trial will assess the efficacy of durvalumab in combination with mitomycin C plus 5-fluorouracil based CRT in patients with locally advanced anal squamous cell carcinoma.

## Conclusion

The mechanisms of carcinogenesis in HPV-related cancers, the unique tumor immune microenvironment, and the effect of radiotherapy on these mechanisms are described. In addition, we discussed the current status of the combination of radiotherapy and immune checkpoint inhibitors, which will be high-lightened.
